# Study of gut microbiota alterations in Alzheimer's dementia patients from Kazakhstan

**DOI:** 10.1038/s41598-022-19393-0

**Published:** 2022-09-06

**Authors:** Aiym Kaiyrlykyzy, Samat Kozhakhmetov, Dmitriy Babenko, Gulnaz Zholdasbekova, Dinara Alzhanova, Farkhad Olzhayev, Aida Baibulatova, Almagul R. Kushugulova, Sholpan Askarova

**Affiliations:** 1 Laboratory of Bioengineering and Regenerative Medicine, Center for Life Sciences, National Laboratory Astana, Nazarbayev University, Nur-Sultan, Kazakhstan; 2 Laboratory of Human Microbiome and Longevity, Center for Life Sciences, National Laboratory Astana, Nazarbayev University, Nur-Sultan, Kazakhstan; 3 Kazakhstan Society of Human Microbiome Researchers, Nur-Sultan, Kazakhstan; 4 Medical University Karaganda, Karagandy, Kazakhstan; 5 Innovative Center ArtScience, Nur-Sultan, Kazakhstan; 6 Graduate School of Public Health, Al-Farabi Kazakh National University, Almaty, Kazakhstan; 7 Department of Neurology, Medical University Astana, Nur-Sultan, Kazakhstan

**Keywords:** Microbiome, Alzheimer's disease

## Abstract

We have investigated the diversity and composition of gut microbiotas isolated from AD (Alzheimer's disease) patients (n = 41) and healthy seniors (n = 43) from Nur-Sultan city (Kazakhstan). The composition of the gut microbiota was characterized by 16S ribosomal RNA sequencing. Our results demonstrated significant differences in bacterial abundance at phylum, class, order, and genus levels in AD patients compared to healthy aged individuals. Relative abundance analysis has revealed increased amount of taxa belonging to *Acidobacteriota, Verrucomicrobiota, Planctomycetota and Synergistota* phyla in AD patients. Among bacterial genera, microbiotas of AD participants were characterized by a decreased amount of *Bifidobacterium, Clostridia bacterium, Castellaniella, Erysipelotrichaceae UCG-003, Roseburia, Tuzzerella, Lactobacillaceae* and *Monoglobus.* Differential abundance analysis determined enriched genera of *Christensenellaceae R-7 group, Prevotella, Alloprevotella, Eubacterium coprostanoligenes group, Ruminococcus, Flavobacterium, Ohtaekwangia, Akkermansia, Bacteroides *sp.* Marseille-P3166* in AD patients, whereas *Levilactobacillus, Lactiplantibacillus, Tyzzerella, Eubacterium siraeum group, Monoglobus, Bacteroides, Erysipelotrichaceae UCG-003, Veillonella, Faecalibacterium, Roseburia, Haemophilus* were depleted. We have also found correlations between some bacteria taxa and blood serum biochemical parameters. Adiponectin was correlated with *Acidimicrobiia*, *Faecalibacterium*, *Actinobacteria*, *Oscillospiraceae*, *Prevotella* and *Christensenellaceae R-7*. The *Christensenellaceae R-7* group and *Acidobacteriota* were correlated with total bilirubin, while *Firmicutes, Acidobacteriales bacterium, Castellaniella alcaligenes, Lachnospiraceae*, *Christensenellaceae* and *Klebsiella pneumoniae* were correlated with the level of CRP in the blood of AD patients. In addition, we report the correlations found between disease severity and certain fecal bacteria. This is the first reported study demonstrating gut microbiota alterations in AD in the Central Asian region.

## Introduction

Alzheimer's disease (AD) is a progressive neurodegenerative disease characterized by memory loss, dramatic changes in character and behavior, and the impossibility of carrying out normal daily activities in the latter stages of the disease. AD incidence increases with age and impacts approximately 10% of people aged 65–75 and 32% of the elderly aged 80 and above^1,2^. As indicated by the World Health Organization (WHO) frequency of AD is exacerbating each year; in this way, it is hypothesized that there could be a triple expansion in the number of AD patients by 2050, and the most significant increase in dementia will occur in low and middle-income countries^3^. In Kazakhstan and other countries of the World, there has been an increase in older adults over the past decades. A recent pilot study on cognitive impairment revealed that the prevalence of mild cognitive impairment (MCI) is about 40% among the elderly population in the Almaty city region, Kazakhstan^4^. Because up to 30% of MCI develops into AD^2^, we can approximate the prevalence of AD in Kazakhstan up to 15% among people aged 65 and over, which is projected to triple by 2050^3^. However, despite the estimated high prevalence, the Kazakh population is not represented in global studies of dementia. The 2015 World Alzheimer's Disease Report states a constant lack of research on dementia in the Central Asian region (the region Kazakhstan belongs to)^3^.

Many risk factors have been recognized as contributors to the development of AD. These include non-modifiable (age, gender, family history, genetics) and modifiable factors such as low education, midlife hypertension, high cholesterol, physical inactivity, obesity, diabetes, etc. The gut microbiota is one of the most important factors influencing human health, and it has gotten a lot of attention from scientists in the last two decades. There are roughly 1000 species and 7000 strains of microscopic organisms that populate the human digestive tract (1013–1014 microorganisms altogether), among which the most widely recognized are *Firmicutes* (51%) and *Bacteroidetes* (48%)^5^. The remaining 1% of bacteria belong to other divisions such as *Proteobacteria, Actinobacteria, Fusobacteria, Spirochaetes, Verrucomicrobia,* and *Lentispherae*^5^. Not long ago, understanding the gut microbiota's role was limited to the processes that entirely occur in the intestine. Nonetheless, the composition of the intestinal microbiota has been studied during the last 15 years, resulting in a direct link between the density and species variety of the gut microbiota and a range of pathological disorders, including diabetes, obesity, and cardiovascular diseases. These disorders, in turn, are well-known risk factors for the development of sporadic AD, and there is data showing that the gut microbiota impacts brain functions^6–8^. Besides, ongoing investigations have uncovered the critical contrasts in the amount and nature of gut microbiota in AD patients compared to healthy seniors of a similar age^9–12^.

For instance, two case–control studies conducted at Wisconsin Alzheimer's infection Research Center (USA)^10^ and Chongqing Medical University (China)^11^ uncovered the differences in intestinal microbiotas composition in patients with AD compared to healthy individuals at the phylum and species levels. Though, personal changes in the intestinal microbiome in Chinese patients varied from those in the United States. These differences may be attributed to biogeography, ethnicity, lifestyle, and eating habits^13^. Therefore, more research is needed to reveal the relationships between microbiota and lifestyle in different ethnic populations and their influence on AD's cognitive functions and risks.

The Asian population is heterogeneous, and microbiota biomarkers in the Kazakhstani population differ from previously published ones, including those from other Asian people^14^. Our previous research has demonstrated that Kazakh microbiotas collected from healthy subjects and metabolic syndrome patients are relatively different from European and East Asian counterparts. This is important for developing prognostics and preventive measures^14^. Yet, no studies have been published on the association between gut microbiota and AD risks in the Central Asian region. Thus, in the present paper, we report the first pilot case–control study of the diversity and composition of gut microbiota isolated from local patients diagnosed with AD compared to healthy seniors. To our knowledge, this is the first study in the Central Asian region investigating gut microbiota biomarkers in association with AD.

## Methods

Forty-one individuals diagnosed with Alzheimer's disease and forty-three cognitively normal controls were recruited from inpatient and outpatient treatment and prevention facilities in Nur-Sultan, Kazakhstan. For case selection, the following inclusion criteria were identified: (a) diagnosis of dementia due to Alzheimer's dementia according to the guidelines for diagnosis and statistics of mental disorders (DSM-IV) and the criteria of the National Institute of Neurological and Communicative Disorders, stroke, Alzheimer's disease and other related disorders (NINCDS-ADRDA)^14^; (b) age from 55 years and older at the time of diagnosis and data collection; (c) voluntary consent to participate in the study. The following inclusion criteria were identified for control selection: (a) absence of cognitive and memory impairment; (b) voluntary consent to participate in the study. We excluded subjects with severe somatic diseases of the kidneys, liver, severe chronic obstructive pulmonary disease, cancer, etc., and subjects with a mental disorder not related to AD.

### Data collection and diagnosis of AD

The experienced neurologists confirmed the diagnosis of Alzheimer's disease according to the criteria of NINCDS-ADRDA (The National Institute of Neurological and Communicative Disorders and the Alzheimer's Disease and Related Disorders Association)^15^. Evaluation of cognitive functions was performed using a mini-mental state examination scale (MMSE) and a clock drawing test (CDT). The MMSE scores were distributed as the following: no dementia (30 points), questionable (26–29), mild and moderate (11–25), and severe dementia (0–10)^16^. Medical history data (history of diabetes, heart disease, hypertension, and any other health condition), complaints, socio-demographic data, and risk factors were collected from research participants and their guardians using questionnaires. The study protocol was approved by the National laboratory Astana Local Ethics Committee. Written informed consent was obtained from all subjects involved in the study.

### Blood sample collection and biochemical analyses

The study participants were sampled fasting venous blood by qualified medical personnel in disposable plastic vacuum tubes with K2-EDTA (purple cap, 10 ml) and coagulation activator gel (yellow hat, 8 ml). Blood samples were left to clot at room temperature for 30 min and then centrifuged for 10 min at 4000 rpm to prepare serum. Determination of the serum levels of lipids was outsourced to the commercial clinical-diagnostic laboratory "Olymp," which routinely performs biochemical analyses. The adiponectin level of the samples was measured using a serum, plasma, and cell culture adiponectin quantification kit (Sigma-Aldrich) according to the manufacturer's protocol.

### Fecal sample collection, DNA isolation, and sequencing

Study participants were given instructions and a special kit for self-collection of feces. The toilet was covered with a white bag labeled "Class A" according to the instructions. A filter paper-sized 9 × 12.5 was then placed in the center before defecation. The feces were placed in a special tube with a spoon, and the tubes were packed in a soft foil envelope and stored in a freezer at – 20 °C until a doctor's visit. Bacterial DNA was isolated according to the QIAamp DNA stool Mini Kit protocol (Qiagen, 51504). Sterile water served as a negative control. Following the standard Illumina protocols, samples were sequenced at Novogene (Beijing, China).

### Sequence analysis

The final 84 samples from AD patients and the healthy group were pooled into four libraries according to 16S rRNA sequencing data. Raw data quality control was carried out using FastQC v0.11.7 programs (Andrews S. et al. FastQC: a quality control tool for high throughput sequence data—2010) и MultiQC v1.8^17^. Sequence data were processed through the LotuS pipeline as previously described^18^ using following parameters: quality filtered (minimum length = 170, minAvgQuality = 27, TruncateSequenceLength = 170, maxAccumulatedError = 0.75) and demultiplexed with sdm (pdiffs = 1, bdiffs = 1). Chimera filtering was undertaken using USEARCH de novo chimera filtering (abundance annotation = 0.97, abskew = 2).

### Statistical analysis

Non-bacterial domain OTUs and OTUs with a total read count of less than 0.001% of the total read count in all samples were removed. All statistical analyses were performed in R v. 4.1.2^19^; graphics were conducted in R using the package ggplot2^20^. Feature table, taxonomic assignment, and tree files for the OTU datasets were imported into phyloseq^21^ for downstream analyses. Alpha diversity metrics such as Shannon^22^, Simpson^23^, Chao1, Observed, ACE and Fisher were calculated with phyloseq on absolute sample abundance values. For ordination plots of beta diversity metrics, sampling counts were first transformed with the Hellinger standardization transformation method^24^. Then weighted Unifrac distance^25^ was calculated, and the graphs of the Principal Coordinate Analysis (PCoA) were generated from a distance^26^. ANOSIM and PERMANOVA tests^27^ with 9999 permutations compared the AD vs. Normal controls. LEfSe was used for differential taxa abundance testing, using default recommended settings according to the author's instructions^28^, at an adjusted p ≤ 0.05 for significance and requiring an LDA effect size of at least 2 for every significant call. Correlation analysis between blood biomarkers (absolute indicators) and OTU (relative indicators) was carried out on the basis of the Spearman test. The list of blood biomarkers included markers that had differences between patients and healthy people (ADPQ, TBIL, TRIG (p < 0.05)), as well as CRP (p = 0.058). The list of OTUs is represented by taxa derived from LEfSe analysis. Also, MaAsLin2 was used to determine specific OTUs associated with disease severity, with correlations considered significant at the 5% level^29^.

### Ethics declarations

The study was conducted in accordance with the Declaration of Helsinki and approved by the Local Ethics Committee of National Laboratory Astana Nazarbayev University (protocol code 05-2020 from 24.09.2020).

## Results

### Analysis of clinical and biochemical data in research groups

The characteristics of the study subjects are presented in Table 1. Individuals with AD had significantly lower MMSE and CDT scores as expected. The frequency of comorbidities such as hypertension, diabetes, and coronary health disease was not statistically different between groups. Body mass index (BMI) was lower in the AD group. Carriers of ApoE ɛ4 were more frequent in the AD group, albeit not statistically significant (possibly, due to the low sample size).

**Table 1:** Study subjects’ characteristics.

Characteristics	AD (n = 41)	Controls (n = 43)	P-value
Age in years, median (IQR)	68 (62–74)	68 (61–75)	0.902
Female, n (%)	30 (73.2%)	35 (81.4%)	0.368
Kazakhs, n (%)	30 (73.2%)	31 (72.1%)	0.866
MMSE score, median (IQR)	15 (6–22)	29 (28–30)	< 0.001
CDT score, median (IQR)	4 (2–5)	9	< 0.001
BMI, median (IQR)	22.7 (21.8–25)	27.4 (24.4–30.1)	< 0.001
Diabetes mellitus, n (%)	9 (22%)	12 (27.9%)	0.528
Hypertension, n (%)	15 (36.6%)	18 (41.8%)	0.620
Coronary heart disease, n (%)	10 (24.4%)	10 (23.3%)	0.902
Carrier of ApoE ɛ4, n (%)^a^	16 (51.6%)	10 (32.3%)	0.123

AD, Alzheimer's disease; MMSE, Mini-mental State Examination scale; CDT, a clock drawing test; BMI, body mass index. Data presented as median (Q1–Q3). Wilcoxon signed-rank test was used to compare medians, and the chi-square test was used to compare frequencies between groups.

^a^For ApoE ɛ4 genotyping sample of 62 (31 in each group) was available.

Also, the analysis did not reveal a statistically significant difference in the blood levels of total cholesterol, low-density lipoprotein (LDL), high-density lipoprotein (HDL), insulin, atherosclerotic index, blood glucose, aspartate aminotransferase (AST), alanine aminotransferase (ALT), and C-reactive protein (CRP) between the compared research groups. This indicates the relative homogeneity of the studied groups. However, levels of triglycerides were identified to be reduced while total bilirubin and serum adiponectin were observed to be eleveated amongst the diseased samples significantly (p < 0.05). (Table 2).

**Table 2:** Comparative characteristics of laboratory parameters (serum concentrations).

Characteristics	AD (n = 41)	Controls (n = 43)	P-value
Serum adiponectin,μg/mL	18.4 (9.8–30.0)	10.8 (5.7–14.5)	0.0069
Fasting glucose, mmol/L	4.8 (4.2–5.2)	4.8 (4.1–5.7)	0.1560
ALT, IU/L	14 (10.4–20.2)	13.9 (11.1–20.9)	0.3851
AST, IU/L	18.5 (15.7–20)	18.6 (15.9–21.8)	0.4604
Total bilirubin, µmol/L	6.8 (5.5–8.7)	5.5 (3.7–7.4)	0.0128
Total cholesterol, mmol/L	4.9 (4.2–5.2)	4.9 (4.3–5.7)	0.5337
HDL, mmol/L	1.3 (1–1.5)	1.1 (1–1.4)	0.2885
LDL, mmol/L	3.2 (2.6–3.7)	3.2 (2.6–3.7)	0.9616
Triglycerides, mmol/L	1.1 (0.9–1.4)	1.5 (0.9–2.4)	0.0094
Insulin, mIU/L	8.1 (6.1–11.2)	11.4 (8–16.8)	0.0565
Atherosclerotic index (LDL/HDL)	2.8 (2.2–3.5)	3.1 (2.5–4.7)	0.0807
C-reactive protein, mg/L	1 (0.5–3.6)	3 (1–4.7)	0.06

ALT, alanine aminotransferase; AST, aspartate aminotransferase; HDL, high-density lipoprotein; LDL, low-density lipoprotein. Data presented as median (Q1–Q3), Wilcoxon signed-rank test was used to compare median frequencies between groups.

### Fecal microbiota analysis

**Figure 1:**
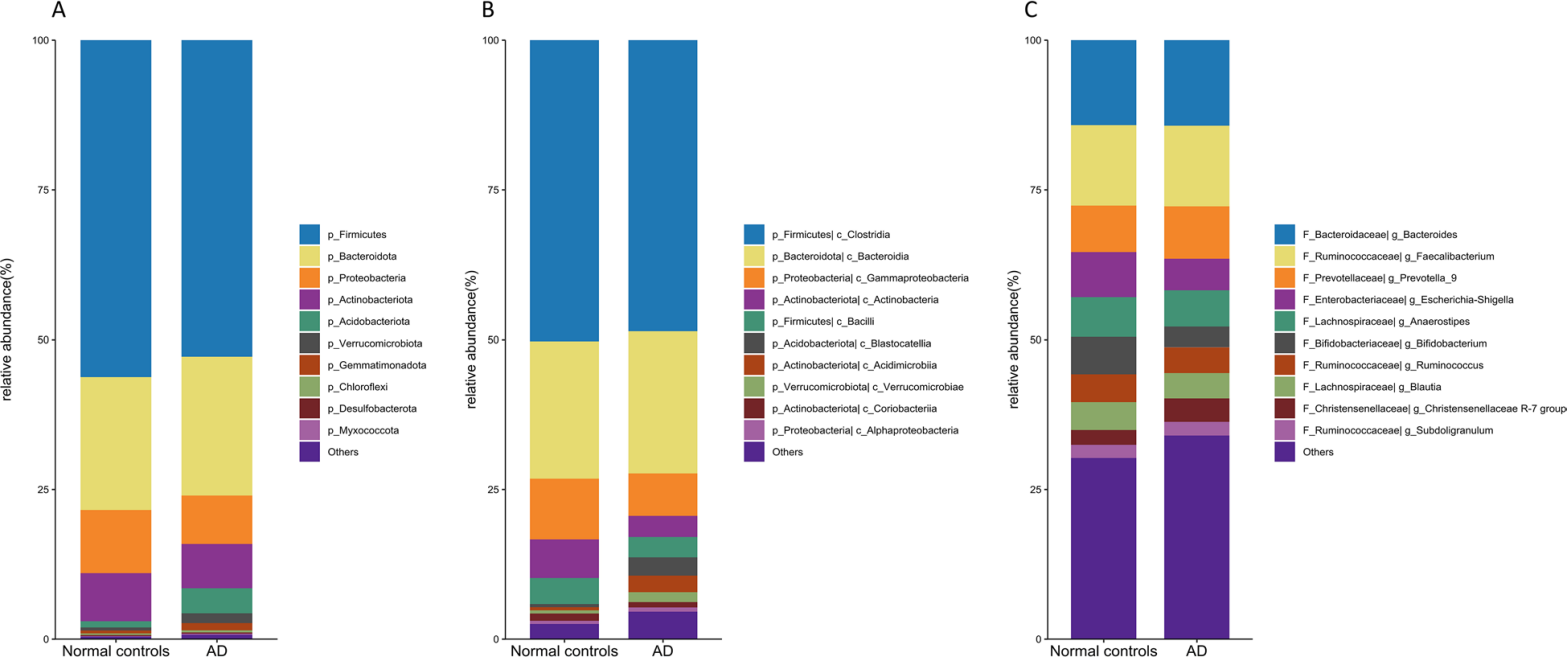
Relative abundance of the bacterial types in the stool samples. Normal control (healthy controls), AD (individuals with Alzheimer's disease). (**A**) Phylum level, (**B**) class level, (**C**) genus level.

**Figure 2:**
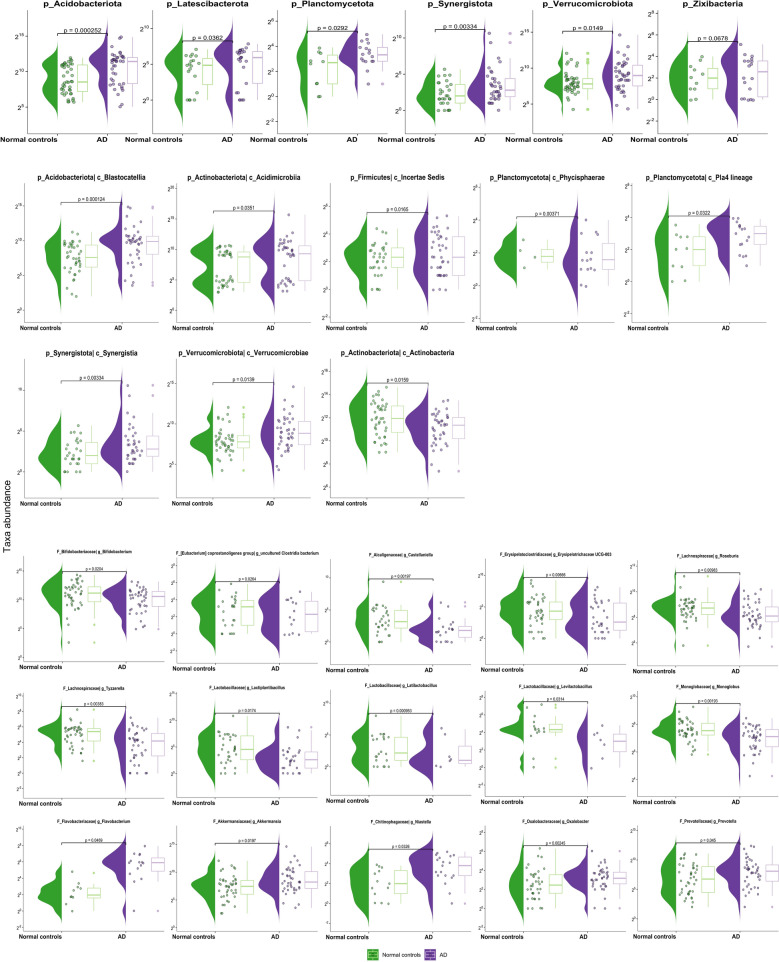
Violin plots of differentially abundant taxa between AD and healthy controls.

To determine the hallmarks of the gut microbiota in Alzheimer's disease, we have analyzed the composition and diversity of bacterial taxa in patients with a confirmed diagnosis versus controls at phylum, class, and genus levels (Figs. 1, 2, Supplement file Table 1). We have found that *Firmicutes, Bacteroidota, Proteobacteria,* and *Actinobacteriota* were the predominant phyla both in AD and controls (Fig. 1A). However, we found higher abundance of *Acidobacteriota* (p < 0.001), *Latescibacterota* (p = 0.036), *Verrucomicrobiota* (p = 0.0149), *Synergistota* (p = 0.0033), *Planctomycetota* (p = 0.0292), and *Zixibacteria* (p = 0.0678) in AD group compared to healthy controls (Fig. 2, Supplement file Table 1).

At class level the predominant bacteria in both groups were *Clostridia, Bacteroidia, Gammaproteobacteria* (Fig. 1B), while amount of *Actinobacteria* (p = 0.0159) were depleted, whereas *Blastocatellia* (p = 0.0001), *Synergistia* (p = 0.0033), *Vicinamibacteria* (p = 0.0139), *lineages Pla4* (p = 0.0322), *Phycishaerae* (p = 0.0037) were enriched in AD group compared to the healthy controls (Figs. 1B, 2). At genus level, the relative abundance of *Bifidobacterium* (p = 0.0204), *uncultured Clostridia bacterium* (p = 0.0264), *Castellaniella* (p = 0.0019*), Erysipelotrichaceae UCG-003* (p = 0.0066), *Roseburia* (p = 0.0098), *Tuzzerella* (p = 0.0038), genus related to family *Lactobacillaceae* (*Lactiplantibacillus, Latilactobacillus, Levilactobacillus* p < 0.05) and *Monoglobus* (p = 0.0019) were decreased in AD patients. At the same time, the microflora in the AD group was enriched with the taxa *Akkermansia* (p = 0.0197), *Niastella* (p = 0.0326), *Oxalobacter* (p = 0.024), *Prevotella* (p = 0.045), *Flavobacterium* (p = 0.0469) (Figs. 1C, 2).

**Figure 3:**
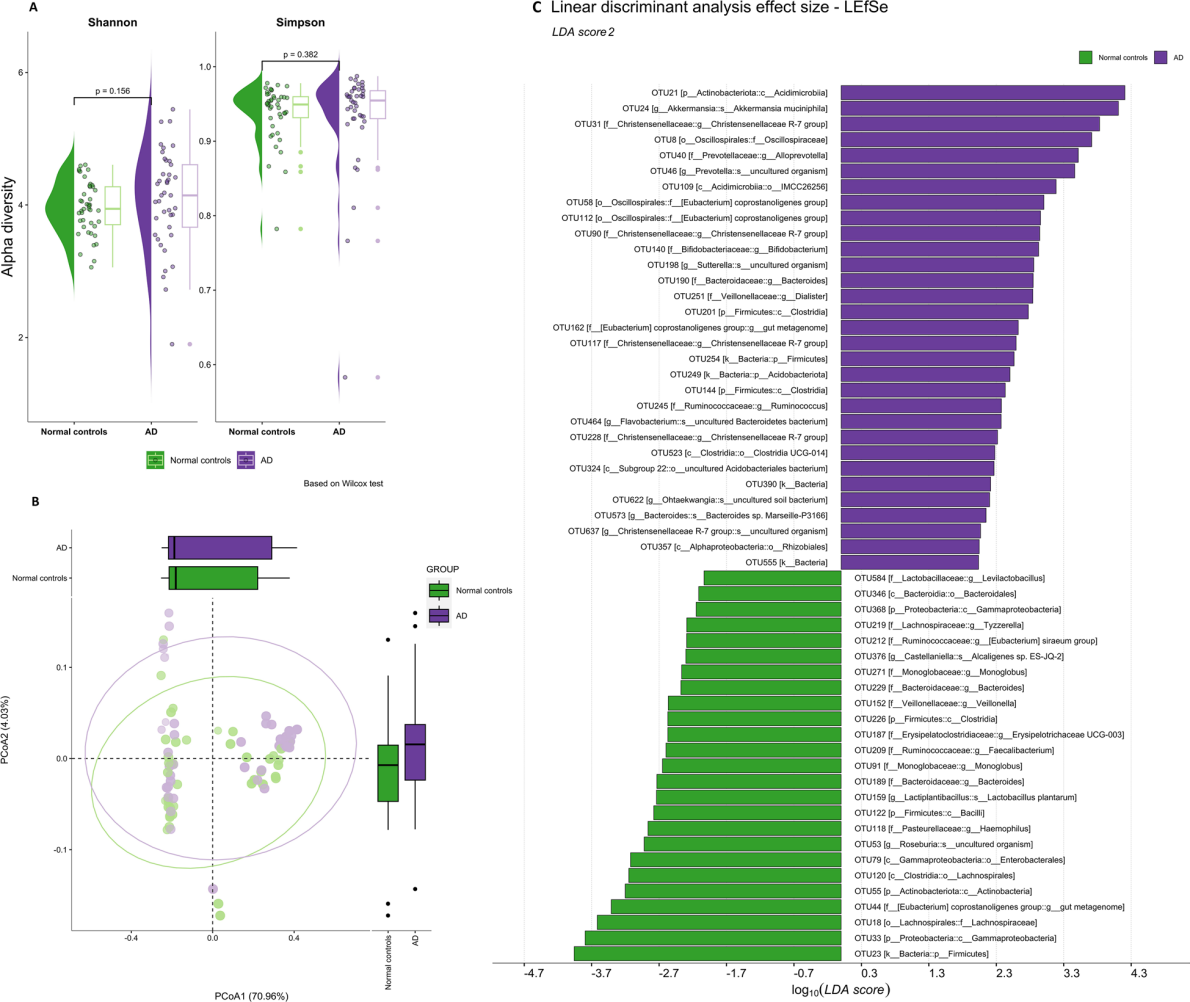
(**A**) α-diversity (Shannon, Simpson indexes) of fecal bacteria in individuals with Alzheimer's disease (AD) and normal controls. (**B**) β-diversity (weighted UniFrac distance) of fecal bacteria in individuals with Alzheimer's disease (AD) and healthy controls (Normal control). (**C**) The linear discriminant analysis (LDA) scores (LEfse plot). An LDA score (log 10) > 2 indicates a significantly different enrichment of bacteria taxa in the AD group (purple) compared to the control group (green). *p* phylum, *c* class, *o* order, *f* family, *g* genus, *s* species.

Analysis of α-diversity using Shannon, Simpson, Chao1, Observed, ACE, and Fisher indices did not reveal any significant differences between AD individuals and healthy controls (Fig. 3A, Supplement file Table 2). Bacterial composition clustering using PCoA of weighted UniFrac distance via Hellinger-transformed data (β-diversity) has also not revealed group-dependent segregation between AD comparing control (p = 0.2313, R^2^ = 0.011) (Fig. 3B). Checking other axes also did not allow us to clearly separate the samples between groups (Supplementary file Fig. 1). The relatively low sample size could explain the absence of statistical significance; therefore, more studies are needed.

Next, we used a linear discriminant analysis effect size (LEfSe) and determined enriched taxa of Genus level *Christensenellaceae R-7 group, Prevotella, Alloprevotella, Bifidobacterium (OTU140), Eubacterium coprostanoligenes group, Ruminococcus, Flavobacterium, Ohtaekwangia, Akkermansia, Bacteroides sp. Marseille-P3166* in AD patients, whereas *Levilactobacillus, Lactiplantibacillus, Tyzzerella, Eubacterium siraeum group, Monoglobus, Bacteroides, Erysipelotrichaceae UCG-003, Veillonella, Faecalibacterium, Roseburia, Haemophilus* were depleted (Fig. 3C). The opposite enrichment direction of *Bifidobacterium* may demonstrate that in patients with AD, the *Bifidobacterium* taxon at the genus level is reduced, while the number of sequences related to *Bifidobacterium* (OTU140) has increased. This discrepancy might be explained by the lower level of the identified taxon, namely, its species affiliation. Comparison of data obtained from two different methods of analysis (LefSe and MaaSlin2) revealed a consistent list of taxa *Faecalibacterium (OTU209), Monoglobus (OTU271), Eubacterium siraeum group (OTU212), Bacteroides (OTU229), Gammaproteobacteria (OTU33)* reduced in the AD group (Supplement file Table 3).

### Correlation between gut microbiota community and blood biochemical parameters

**Figure 4:**
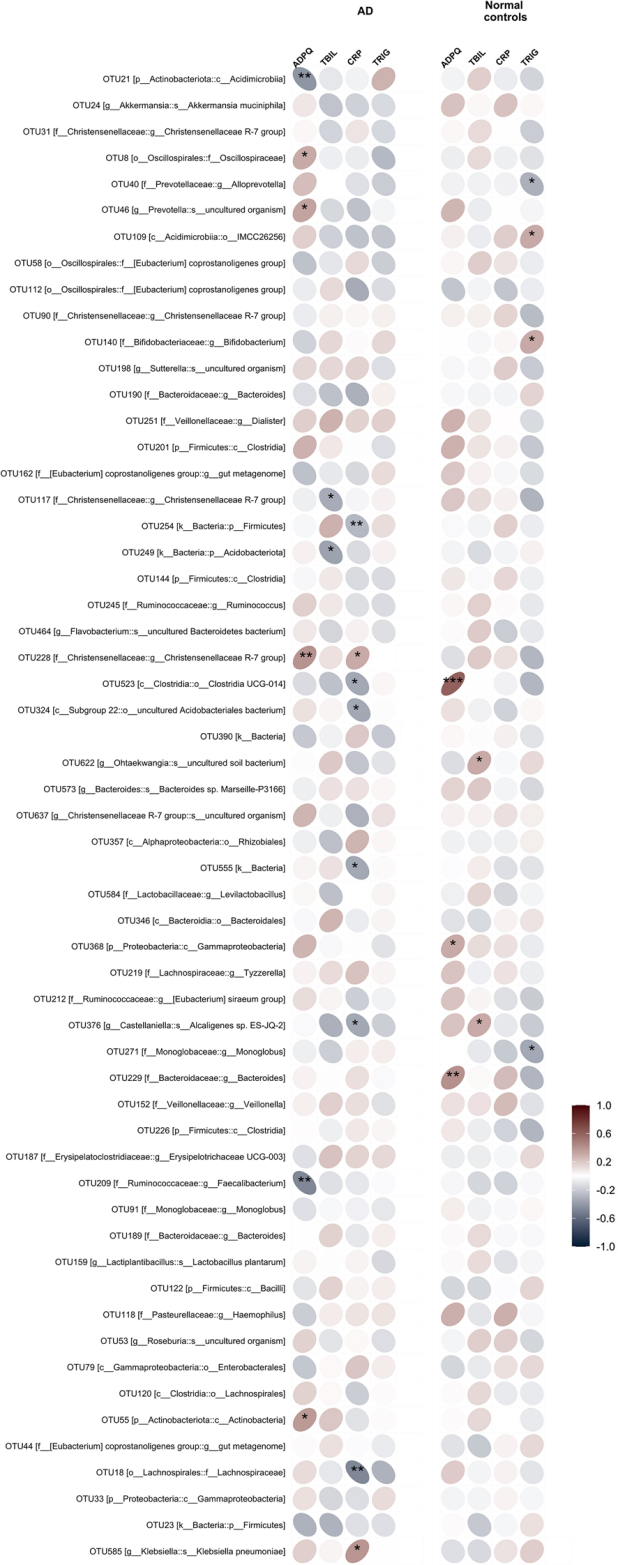
Correlation between adiponectin, C-reactive protein, total bilirubin, triglycerides, and differential bacterial taxa in AD and controls. *ADPQ* adiponectin, *TBIL* total bilirubin, *CRP* C-reactive protein, *TRIG* triglycerides.

We investigated possible associations between the blood laboratory parameters and gut microbiota composition in AD patients and healthy aged individuals using correlation analysis between serum adiponectin, cholesterol, HDL, LDL, triglycerides, insulin, fasting glucose, ALT, AST, total bilirubin, C-reactive protein and different bacterial taxa found in control and AD groups (Fig. 4). In the AD group, we have seen a negative correlation of adiponectin with *Acidimicrobiia* (OTU21) at the class level and *Faecalibacterium* (OTU209) at the genus level. We have also observed positive correlations of adiponectin with *Actinobacteria* (OTU55), *Oscillospiraceae* (OTU8), *Prevotella* (OTU46) and *Christensenellaceae R-7* group (OTU228). The *Christensenellaceae R-7* group (OTU117) and *Acidobacteriota* (OTU249) were negatively correlated with total bilirubin. *Firmicutes* (OTU254), *Acidobacteriales bacterium* (OTU324), indeterminate taxon (OTU555), *Castellaniella alcaligenes* (OTU376), *Lachnospiraceae* family taxon (OTU18) were negatively correlated with CRP, while *Christensenellaceae R-7* (OTU228) and *Klebsiella pneumoniae* (OTU585) were positively correlated with the level of CRP in the blood of AD patients. In controls, *Bacteroides* (OTU229)*, Gammaproteobacteria* (OTU368)*,* and *Clostridia UCG-014* (OTU523) were positively correlated with blood levels of adiponectin. Blood bilirubin levels were positively correlated with *Castellaniella alcaligenes* (OTU376) and uncultured *Ohtaekwangia* (OTU622). The taxa *Acidimicrobiia* (OTU109) and *Bifidobacterium* (OTU140) were positively correlated while the taxa *Alloprevotella* (OTU40) and *Monoglobus* (OTU271) were negatively correlated with the level of triglycerides in control.

### Correlation between gut microbiota community and disease severity

The moderate form of AD was correlated with *Bacteroides* (OTU3880), *Methylomirabilota* (OTU903), uncultured *Clostridiales bacterium* (OTU2480), *Prevotellaceae* (OTU726), and the severe AD was correlated with *Symbiobacteraceae* (OTU530), *uncultured proteobacterium* (OTU3544), *Clostridia vadinBB60 group* (OTU406), *Collinsella* (OTU2030), *Caldilineales* (OTU300), *Latescibacterota uncultured prokaryote* (OTU3172), *Elusimicrobiota Lineage Iib uncultured archaeon* (OTU5211), *Christensenellaceae R-7 group uncultured Clostridia bacterium* (OTU1726) and *gut metagenome* (OTU1452) (Supplement file Table 4).

## Discussion

In the past decade, numerous studies on the relationship between the microbiota and the central nervous system (CNS) have been published, suggesting the conception of the "brain-gut-microbiota axis"^8^. There are a lot of published papers showing the impact of intestinal dysbiosis, caused by changes in diet, antibiotics, non-steroidal anti-inflammatory drugs, presence of pathogenic microorganisms on the cognitive capacities of the brain^30,31^. Furthermore, recent research reveals correlations between the number and quality of the gut microbiota and Alzheimer's disease^9–11^.

For instance, studies from animal models of AD revealed substantial differences in gut microbiota composition of transgenic mice expressing the human APP gene and PS1 from wild-type mice^32^. The number of *Firmicutes*, *Verrucomicrobia*, *Proteobacteria,* and *Actinobacteria* was significantly decreased, whereas *Bacteroidetes* and *Tenericutes* were increased in 8-month-old CONVR-APPPS1 mice compared to wildlife mice. Furthermore, in the brain of CONVR-APPPS1 mice bred under sterile conditions, there was a substantial decrease in Aβ deposits relative to animals of the same genotype generated under normal conditions. Furthermore, the level of Aβ expression was upregulated after the microbiota transplantation from the CONVR-APPPS1 mice bred under normal conditions to the CONVR-APPPS1 mice bred in an aseptic environment. In contrast, fecal transplantation from wild-type mice did not change Aβ levels in the CNS.

Evidence obtained from clinical studies verified study results on laboratory animals. For instance, the relationship of brain amyloidosis with intestinal bacterial taxa and peripheral markers facilitating inflammation in people of advanced age with dementia was described^12^. This examination showed that in dementia patients with amyloidosis, an expanded degree of blood pro-inflammatory cytokines (IL-6, CXCL2, NLRP3, and IL-1β) was positively correlated with a number of some pro-inflammatory gut bacteria such as *Escherichia/Shigella* and negatively associated with anti-inflammatory *E.rectale* taxon. These associations of pro-inflammatory gut bacteria with increased levels of pro-inflammatory cytokines in the blood suggest that the alterations of the gut microbiota might be one of the factors responsible for the chronic peripheral inflammation leading to the development of neuroinflammation and neurodegeneration. In support of this notion, studies of microbiota from PD (Parkinson's disease) patients have demonstrated that clinical phenotype and severity of the illness were somehow correlated with the alterations of the gut microbiota and elevated plasma cytokines^33^.

At the Alzheimer's Disease Research Center (Wisconsin Alzheimer's Infection Research Center, USA), researchers discovered significant differences in the composition of the gut microbiota in patients with AD and healthy individuals at the phylum and species levels^10^. In the intestinal microbiota of AD patients, these investigations found a decrease in the number of bacteria belonging to the *Firmicute*s and *Actinobacteria* phyla (particularly bacteria of the genus *Bifidobacterium*) and an increase in the number of bacteria belonging to the *Bacteroidetes* phylum. Furthermore, a differential connection appeared between the degrees of individual bacterial genera in the digestive tract and cerebrospinal markers of AD, for example, Aβ42/Aβ40, p-tau, just as the Aβ/p-tau proportion^10^. *Bacteroides, Actinobacteria, Ruminococcus, Lachnospiraceae*, and *Selenomonadales* were found to have substantial changes in the spectrum of bacteria present in the bowels of individuals with Alzheimer's disease, according to research conducted at Chongqing Medical University in China^11^. Though, personal changes in the intestinal microbiota in Chinese patients varied to some degree from those in the United States. According to Zhuang et al., the number of bacteria belonging to the phylum *Bacteroidetes* is decreased. In contrast, if compared to healthy controls, the number of bacteria in the phylum *Firmicutes* remained unchanged.

In our cohort, differences in bacterial taxa between AD and controls were characterized by the elevated abundance of *Acidobacteriota*, *Latescibacterota*, *Verrucomicrobiota*, *Synergistota, Planctomycetota*, and *Zixibacteria* at the phylum level. Our results also demonstrated a relatively lower abundance of *Bifidobacterium* and microorganisms belonging to a family of *Lactobacillaceae.* Thus, microbiota biomarkers of AD patients from Kazakhstani population differ from previously published reports. However, despite the close geographical location of Kazakhstan to China and the predominantly Asian origin of the Kazakh population, our data are more consistent with the results of Vogt et al.^10^ in comparison with findings of Zhuang et al.^11^, who reported an increased amount of *Lactobacillaceae* and did not report any changes of *Bifidobacterium.* These similarities could result from such factors as the multi-ethnicity of the Kazakh population, westernized eating preferences, and relatively similar climatic conditions of the Nur-Sultan city region and Wisconsin^13^. Interestingly, *Bifidobacteria and Lactobacillaceae* taxa are involved in the production of important metabolites (neurotransmitters, neuroactive metabolites, SCFA, etc.) that play a crucial role in maintaining healthy cognitive, neuropsychiatric function and the reduction of which may be associated with the risk of developing neurodegenerative disorders^34^.

Gut microbiota has been suggested to be actively involved in host metabolism. In particular, several studies have shown that fecal bacteria can influence the metabolism of fatty acids and lipids^35,36^ and affect the expression of adiponectin^37^, a hormone produced by white adipose tissue. For instance, in human studies, the family *Clostridiaceae/Lachnospiracease* was associated with LDL, *Pasteurellaceae, Coprococcus,* and genus C*ollinsella,* and species *Stercoris* showed association with triglyceride levels^38^. The gut composition of individuals with hypercholesterolemia was characterized by a lower abundance of the genera *Anaeroplasma* and *Haemophilus,* while a higher presence of *Odoribacter; Anaeroplasma,* and *Haemophilus* were correlated with lipid profile^39^. The effect of intestinal microbiota on adiponectin expression has been shown in animal models using a high-fat diet in obese mice. Significant correlations have been revealed between lipid metabolism, adiponectin, and altered gut microflora in coronary heart disease^37,40^.

As mentioned, adiponectin is a protein hormone produced mainly by white adipose tissue that regulates insulin sensitivity, fatty acid catabolism, glucose homeostasis, and anti-inflammatory system through various mechanisms. Adiponectin is involved in the pathogenesis of several age-related diseases, such as atherosclerosis, type 2 diabetes, and cardiovascular disorders^41^. In the past ten years, considerable information has been accumulated on the importance of adiponectin activity in the pathogenesis of Alzheimer's disease^42^. According to our findings, the adiponectin level was as twice as elevated in the AD group. This is in line with several studies that demonstrated that the plasma concentration of adiponectin was elevated in patients with mild cognitive impairment (MCI) and AD^43,44^. In our sample of AD patients, elevated levels of serum adiponectin were strongly correlated with *Actinobacteria*, *Acidomicrobiia* at the class level, *Prevotella, Faecalibacterium,* and *Christensenellaceae R-7* at the genus level, and *Oscillospiraceae* at the family level; however, further studies are needed to investigate if this correlation is a result of a causal relationship.

Over the last decade, it become evident that chronic peripheral inflammation is one of the important factors contributing significantly to the development and progression of AD. There is also an assumption that the development of sporadic AD might be driven by the microbiome-associated peripheral inflammation^45^. Previous studies investigating the links between gut microbiota and low-grade inflammation marker C-reactive protein found *Ruminococcaceae, Akkermansia,* and *Lactobacillales* to be associated with the risk of anxiety and depression^46^. In cardiovascular patients, peripheral C-reactive protein was linked to the abundance of *Bifidobacterium, Faecalibacterium, Ruminococcus,* and *Prevotella*^47^. In our study, we observed a correlation between CRP and *Firmicutes, Acidobacteriales bacterium, Castellaniella alcaligenes, Lachnospiraceae, Christensenellaceae* R-7, and *Klebsiella pneumoniae* among the AD group participants.

In conclusion, we have investigated gut microbiota composition in AD patients from the Central Asian region for the first time. Consistent with previous reports, our study has demonstrated that the gut microbiota is altered in individuals with AD. However, microbiota biomarkers of AD patients from Kazakhstan are not identical to microbiomes of AD individuals from other countries. Furthermore, we reported the correlations exciting between blood serum profile and some fecal bacteria taxa. Significant results were also obtained by correlation analysis between the severity of the disease and meta-omics features. However, there are several limitations in our study worth mentioning. The relatively low sample size is one of the limitations of the study, yet comparable with sample sizes in similar published papers. Besides, we have utilized well established 16S ribosomal RNA MiSeq sequencing in our study, although metagenomics sequencing instead of targeted amplicon sequencing is more preferable since they provide broader and more accurate microbial information. By focusing on the mechanism for the interactions between human microbiota, peripheral metabolism, and the brain, we can uncover new pathophysiological pathways leading to the onset and progression of AD and other neurodegenerative disorders. Studying the relationships between human microbiotas and AD in different ethnic populations will help evaluate the contribution of biogeography and lifestyle to the increase in the prevalence of dementia and develop practical recommendations for the prevention and treatment of this severe pathology. However, more study is needed to ascertain our findings and disclose new associations between lifestyle, gut microbiota, and neurodegenerative disorders in different ethnic populations.

## Supplementary Information

Supplementary Information.

## Data Availability

The datasets presented in this study can be found in online repositories. The names of the repository/repositories and accession number(s) can be found below: NCBI BioProject [accession number PRJNA811324].
