# Supplementary material for: Study of gut microbiota alterations in Alzheimer's dementia patients from Kazakhstan
Source: Sci Rep. 2022 Sep 6;12:15115. doi: 10.1038/s41598-022-19393-0 (PMC9448737; doi:10.1038/s41598-022-19393-0)
Supplement: Supplementary file 1 — Supplementary Information. [file 41598_2022_19393_MOESM1_ESM.pptx]

## Slide 1
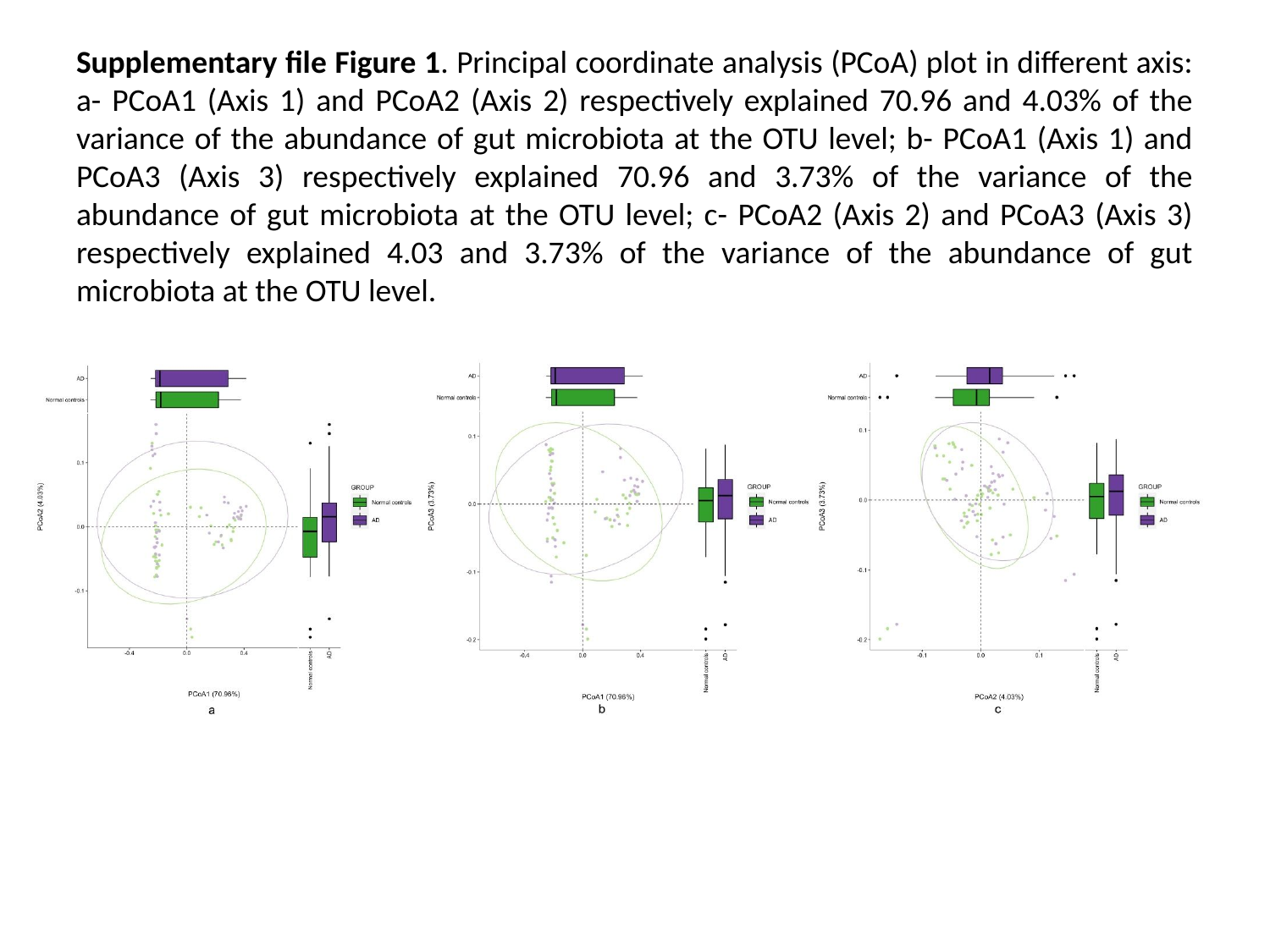

# Supplementary file Figure 1. Principal coordinate analysis (PCoA) plot in different axis: a- PCoA1 (Axis 1) and PCoA2 (Axis 2) respectively explained 70.96 and 4.03% of the variance of the abundance of gut microbiota at the OTU level; b- PCoA1 (Axis 1) and PCoA3 (Axis 3) respectively explained 70.96 and 3.73% of the variance of the abundance of gut microbiota at the OTU level; c- PCoA2 (Axis 2) and PCoA3 (Axis 3) respectively explained 4.03 and 3.73% of the variance of the abundance of gut microbiota at the OTU level.

## Slide 2
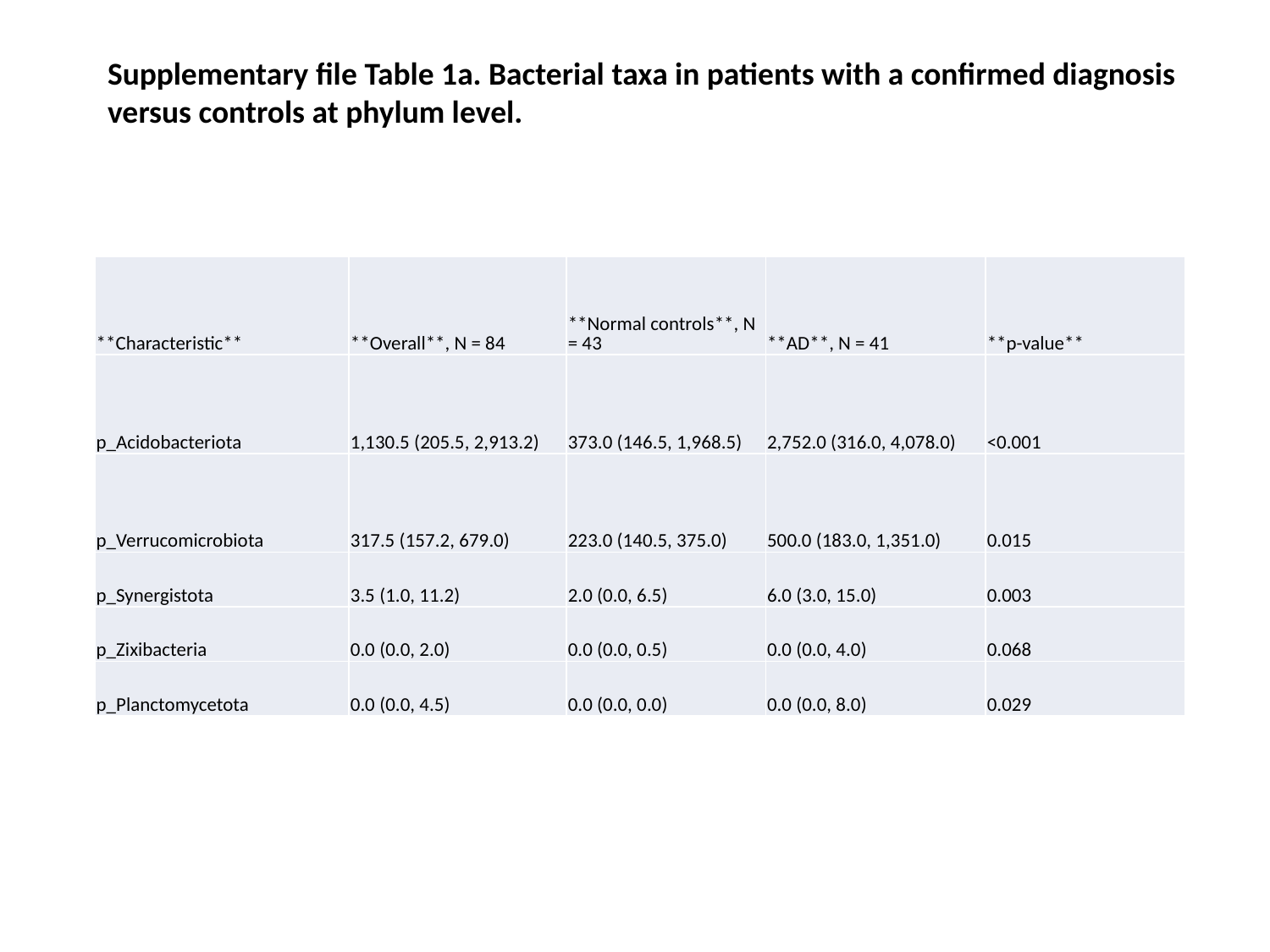

# Supplementary file Table 1a. Bacterial taxa in patients with a confirmed diagnosis versus controls at phylum level.
| \*\*Characteristic\*\* | \*\*Overall\*\*, N = 84 | \*\*Normal controls\*\*, N = 43 | \*\*AD\*\*, N = 41 | \*\*p-value\*\* |
| --- | --- | --- | --- | --- |
| p\_Acidobacteriota | 1,130.5 (205.5, 2,913.2) | 373.0 (146.5, 1,968.5) | 2,752.0 (316.0, 4,078.0) | <0.001 |
| p\_Verrucomicrobiota | 317.5 (157.2, 679.0) | 223.0 (140.5, 375.0) | 500.0 (183.0, 1,351.0) | 0.015 |
| p\_Synergistota | 3.5 (1.0, 11.2) | 2.0 (0.0, 6.5) | 6.0 (3.0, 15.0) | 0.003 |
| p\_Zixibacteria | 0.0 (0.0, 2.0) | 0.0 (0.0, 0.5) | 0.0 (0.0, 4.0) | 0.068 |
| p\_Planctomycetota | 0.0 (0.0, 4.5) | 0.0 (0.0, 0.0) | 0.0 (0.0, 8.0) | 0.029 |

## Slide 3
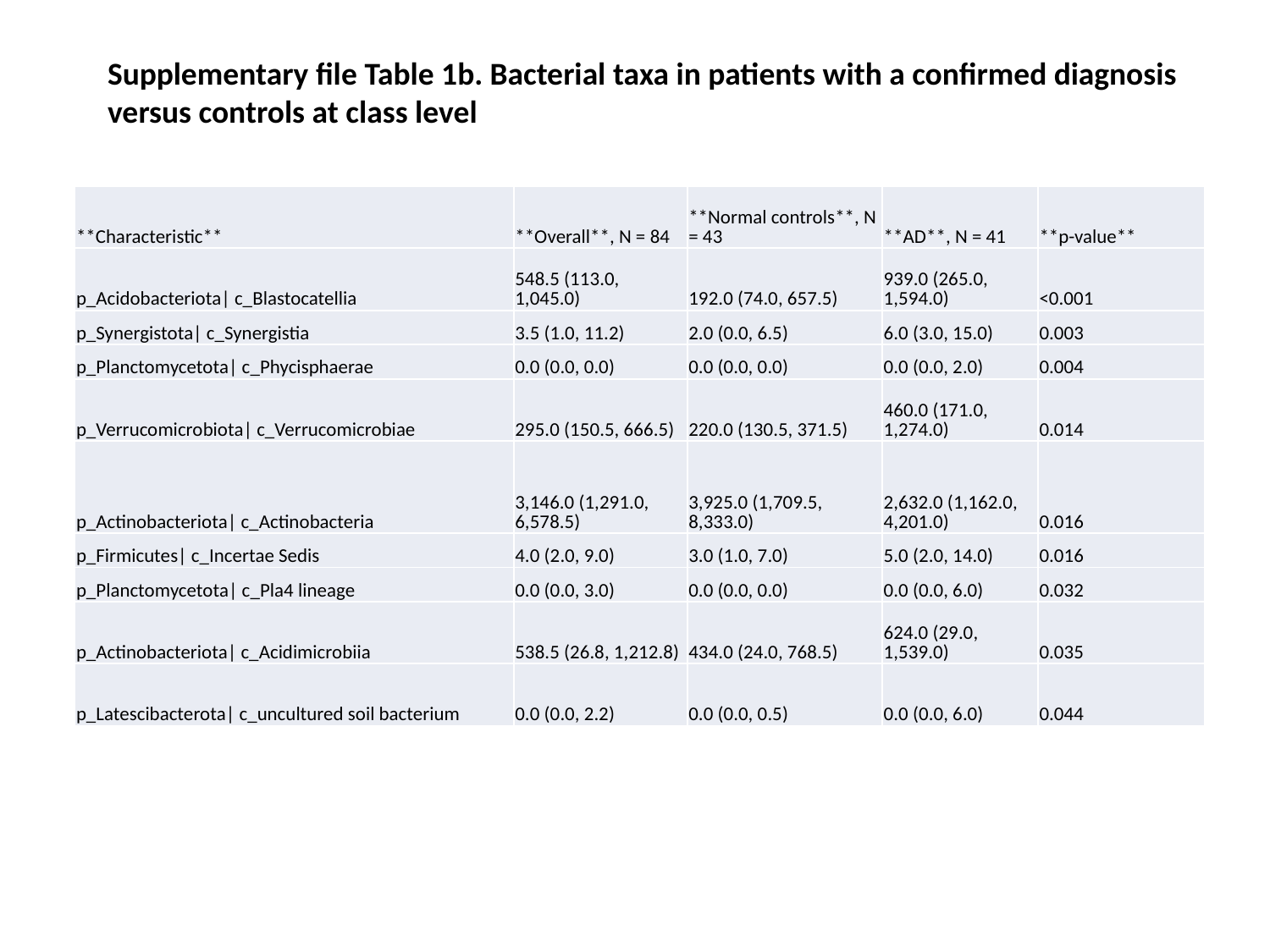

# Supplementary file Table 1b. Bacterial taxa in patients with a confirmed diagnosis versus controls at class level
| \*\*Characteristic\*\* | \*\*Overall\*\*, N = 84 | \*\*Normal controls\*\*, N = 43 | \*\*AD\*\*, N = 41 | \*\*p-value\*\* |
| --- | --- | --- | --- | --- |
| p\_Acidobacteriota| c\_Blastocatellia | 548.5 (113.0, 1,045.0) | 192.0 (74.0, 657.5) | 939.0 (265.0, 1,594.0) | <0.001 |
| p\_Synergistota| c\_Synergistia | 3.5 (1.0, 11.2) | 2.0 (0.0, 6.5) | 6.0 (3.0, 15.0) | 0.003 |
| p\_Planctomycetota| c\_Phycisphaerae | 0.0 (0.0, 0.0) | 0.0 (0.0, 0.0) | 0.0 (0.0, 2.0) | 0.004 |
| p\_Verrucomicrobiota| c\_Verrucomicrobiae | 295.0 (150.5, 666.5) | 220.0 (130.5, 371.5) | 460.0 (171.0, 1,274.0) | 0.014 |
| p\_Actinobacteriota| c\_Actinobacteria | 3,146.0 (1,291.0, 6,578.5) | 3,925.0 (1,709.5, 8,333.0) | 2,632.0 (1,162.0, 4,201.0) | 0.016 |
| p\_Firmicutes| c\_Incertae Sedis | 4.0 (2.0, 9.0) | 3.0 (1.0, 7.0) | 5.0 (2.0, 14.0) | 0.016 |
| p\_Planctomycetota| c\_Pla4 lineage | 0.0 (0.0, 3.0) | 0.0 (0.0, 0.0) | 0.0 (0.0, 6.0) | 0.032 |
| p\_Actinobacteriota| c\_Acidimicrobiia | 538.5 (26.8, 1,212.8) | 434.0 (24.0, 768.5) | 624.0 (29.0, 1,539.0) | 0.035 |
| p\_Latescibacterota| c\_uncultured soil bacterium | 0.0 (0.0, 2.2) | 0.0 (0.0, 0.5) | 0.0 (0.0, 6.0) | 0.044 |

## Slide 4
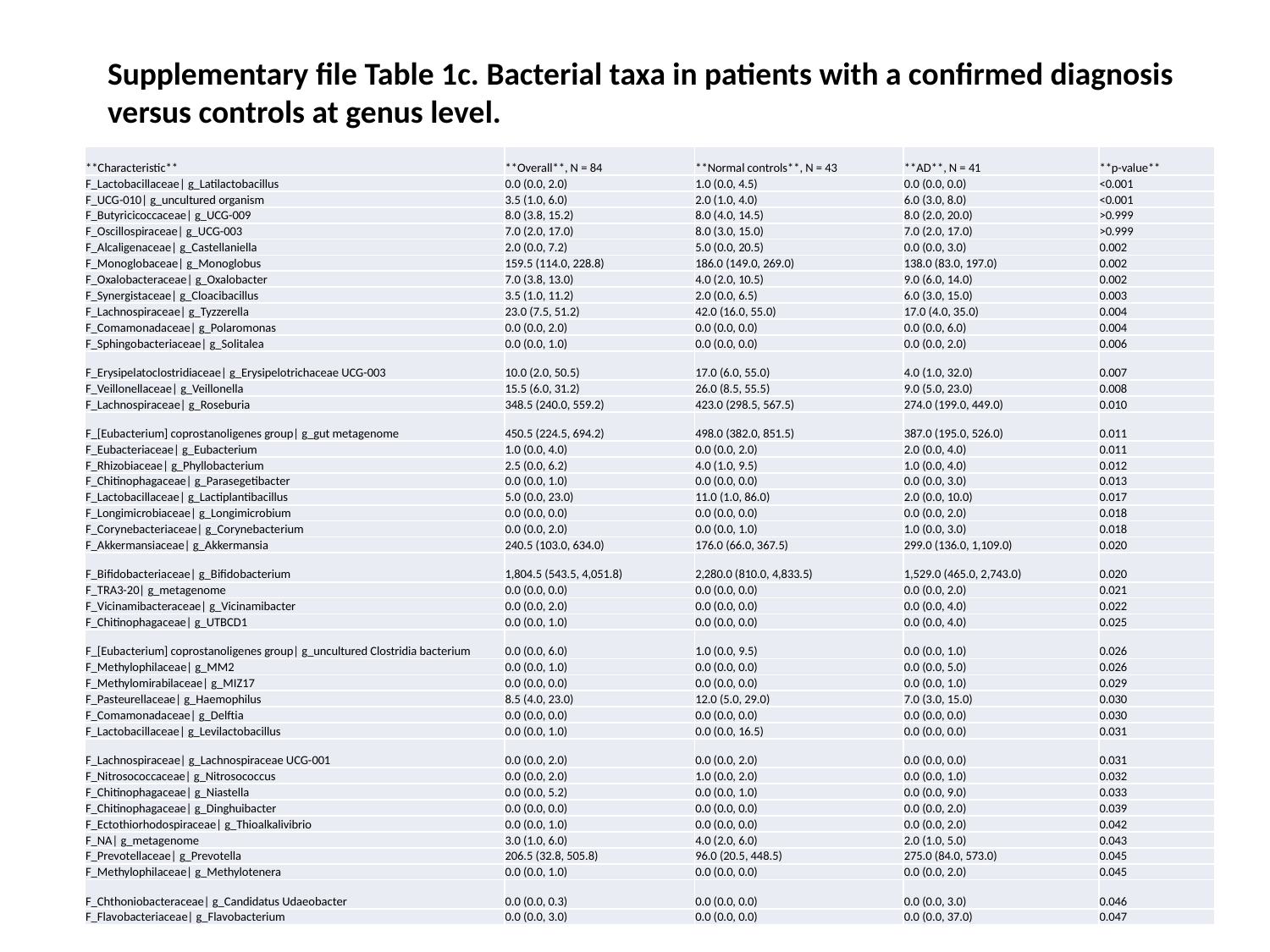

# Supplementary file Table 1c. Bacterial taxa in patients with a confirmed diagnosis versus controls at genus level.
| \*\*Characteristic\*\* | \*\*Overall\*\*, N = 84 | \*\*Normal controls\*\*, N = 43 | \*\*AD\*\*, N = 41 | \*\*p-value\*\* |
| --- | --- | --- | --- | --- |
| F\_Lactobacillaceae| g\_Latilactobacillus | 0.0 (0.0, 2.0) | 1.0 (0.0, 4.5) | 0.0 (0.0, 0.0) | <0.001 |
| F\_UCG-010| g\_uncultured organism | 3.5 (1.0, 6.0) | 2.0 (1.0, 4.0) | 6.0 (3.0, 8.0) | <0.001 |
| F\_Butyricicoccaceae| g\_UCG-009 | 8.0 (3.8, 15.2) | 8.0 (4.0, 14.5) | 8.0 (2.0, 20.0) | >0.999 |
| F\_Oscillospiraceae| g\_UCG-003 | 7.0 (2.0, 17.0) | 8.0 (3.0, 15.0) | 7.0 (2.0, 17.0) | >0.999 |
| F\_Alcaligenaceae| g\_Castellaniella | 2.0 (0.0, 7.2) | 5.0 (0.0, 20.5) | 0.0 (0.0, 3.0) | 0.002 |
| F\_Monoglobaceae| g\_Monoglobus | 159.5 (114.0, 228.8) | 186.0 (149.0, 269.0) | 138.0 (83.0, 197.0) | 0.002 |
| F\_Oxalobacteraceae| g\_Oxalobacter | 7.0 (3.8, 13.0) | 4.0 (2.0, 10.5) | 9.0 (6.0, 14.0) | 0.002 |
| F\_Synergistaceae| g\_Cloacibacillus | 3.5 (1.0, 11.2) | 2.0 (0.0, 6.5) | 6.0 (3.0, 15.0) | 0.003 |
| F\_Lachnospiraceae| g\_Tyzzerella | 23.0 (7.5, 51.2) | 42.0 (16.0, 55.0) | 17.0 (4.0, 35.0) | 0.004 |
| F\_Comamonadaceae| g\_Polaromonas | 0.0 (0.0, 2.0) | 0.0 (0.0, 0.0) | 0.0 (0.0, 6.0) | 0.004 |
| F\_Sphingobacteriaceae| g\_Solitalea | 0.0 (0.0, 1.0) | 0.0 (0.0, 0.0) | 0.0 (0.0, 2.0) | 0.006 |
| F\_Erysipelatoclostridiaceae| g\_Erysipelotrichaceae UCG-003 | 10.0 (2.0, 50.5) | 17.0 (6.0, 55.0) | 4.0 (1.0, 32.0) | 0.007 |
| F\_Veillonellaceae| g\_Veillonella | 15.5 (6.0, 31.2) | 26.0 (8.5, 55.5) | 9.0 (5.0, 23.0) | 0.008 |
| F\_Lachnospiraceae| g\_Roseburia | 348.5 (240.0, 559.2) | 423.0 (298.5, 567.5) | 274.0 (199.0, 449.0) | 0.010 |
| F\_[Eubacterium] coprostanoligenes group| g\_gut metagenome | 450.5 (224.5, 694.2) | 498.0 (382.0, 851.5) | 387.0 (195.0, 526.0) | 0.011 |
| F\_Eubacteriaceae| g\_Eubacterium | 1.0 (0.0, 4.0) | 0.0 (0.0, 2.0) | 2.0 (0.0, 4.0) | 0.011 |
| F\_Rhizobiaceae| g\_Phyllobacterium | 2.5 (0.0, 6.2) | 4.0 (1.0, 9.5) | 1.0 (0.0, 4.0) | 0.012 |
| F\_Chitinophagaceae| g\_Parasegetibacter | 0.0 (0.0, 1.0) | 0.0 (0.0, 0.0) | 0.0 (0.0, 3.0) | 0.013 |
| F\_Lactobacillaceae| g\_Lactiplantibacillus | 5.0 (0.0, 23.0) | 11.0 (1.0, 86.0) | 2.0 (0.0, 10.0) | 0.017 |
| F\_Longimicrobiaceae| g\_Longimicrobium | 0.0 (0.0, 0.0) | 0.0 (0.0, 0.0) | 0.0 (0.0, 2.0) | 0.018 |
| F\_Corynebacteriaceae| g\_Corynebacterium | 0.0 (0.0, 2.0) | 0.0 (0.0, 1.0) | 1.0 (0.0, 3.0) | 0.018 |
| F\_Akkermansiaceae| g\_Akkermansia | 240.5 (103.0, 634.0) | 176.0 (66.0, 367.5) | 299.0 (136.0, 1,109.0) | 0.020 |
| F\_Bifidobacteriaceae| g\_Bifidobacterium | 1,804.5 (543.5, 4,051.8) | 2,280.0 (810.0, 4,833.5) | 1,529.0 (465.0, 2,743.0) | 0.020 |
| F\_TRA3-20| g\_metagenome | 0.0 (0.0, 0.0) | 0.0 (0.0, 0.0) | 0.0 (0.0, 2.0) | 0.021 |
| F\_Vicinamibacteraceae| g\_Vicinamibacter | 0.0 (0.0, 2.0) | 0.0 (0.0, 0.0) | 0.0 (0.0, 4.0) | 0.022 |
| F\_Chitinophagaceae| g\_UTBCD1 | 0.0 (0.0, 1.0) | 0.0 (0.0, 0.0) | 0.0 (0.0, 4.0) | 0.025 |
| F\_[Eubacterium] coprostanoligenes group| g\_uncultured Clostridia bacterium | 0.0 (0.0, 6.0) | 1.0 (0.0, 9.5) | 0.0 (0.0, 1.0) | 0.026 |
| F\_Methylophilaceae| g\_MM2 | 0.0 (0.0, 1.0) | 0.0 (0.0, 0.0) | 0.0 (0.0, 5.0) | 0.026 |
| F\_Methylomirabilaceae| g\_MIZ17 | 0.0 (0.0, 0.0) | 0.0 (0.0, 0.0) | 0.0 (0.0, 1.0) | 0.029 |
| F\_Pasteurellaceae| g\_Haemophilus | 8.5 (4.0, 23.0) | 12.0 (5.0, 29.0) | 7.0 (3.0, 15.0) | 0.030 |
| F\_Comamonadaceae| g\_Delftia | 0.0 (0.0, 0.0) | 0.0 (0.0, 0.0) | 0.0 (0.0, 0.0) | 0.030 |
| F\_Lactobacillaceae| g\_Levilactobacillus | 0.0 (0.0, 1.0) | 0.0 (0.0, 16.5) | 0.0 (0.0, 0.0) | 0.031 |
| F\_Lachnospiraceae| g\_Lachnospiraceae UCG-001 | 0.0 (0.0, 2.0) | 0.0 (0.0, 2.0) | 0.0 (0.0, 0.0) | 0.031 |
| F\_Nitrosococcaceae| g\_Nitrosococcus | 0.0 (0.0, 2.0) | 1.0 (0.0, 2.0) | 0.0 (0.0, 1.0) | 0.032 |
| F\_Chitinophagaceae| g\_Niastella | 0.0 (0.0, 5.2) | 0.0 (0.0, 1.0) | 0.0 (0.0, 9.0) | 0.033 |
| F\_Chitinophagaceae| g\_Dinghuibacter | 0.0 (0.0, 0.0) | 0.0 (0.0, 0.0) | 0.0 (0.0, 2.0) | 0.039 |
| F\_Ectothiorhodospiraceae| g\_Thioalkalivibrio | 0.0 (0.0, 1.0) | 0.0 (0.0, 0.0) | 0.0 (0.0, 2.0) | 0.042 |
| F\_NA| g\_metagenome | 3.0 (1.0, 6.0) | 4.0 (2.0, 6.0) | 2.0 (1.0, 5.0) | 0.043 |
| F\_Prevotellaceae| g\_Prevotella | 206.5 (32.8, 505.8) | 96.0 (20.5, 448.5) | 275.0 (84.0, 573.0) | 0.045 |
| F\_Methylophilaceae| g\_Methylotenera | 0.0 (0.0, 1.0) | 0.0 (0.0, 0.0) | 0.0 (0.0, 2.0) | 0.045 |
| F\_Chthoniobacteraceae| g\_Candidatus Udaeobacter | 0.0 (0.0, 0.3) | 0.0 (0.0, 0.0) | 0.0 (0.0, 3.0) | 0.046 |
| F\_Flavobacteriaceae| g\_Flavobacterium | 0.0 (0.0, 3.0) | 0.0 (0.0, 0.0) | 0.0 (0.0, 37.0) | 0.047 |

## Slide 5
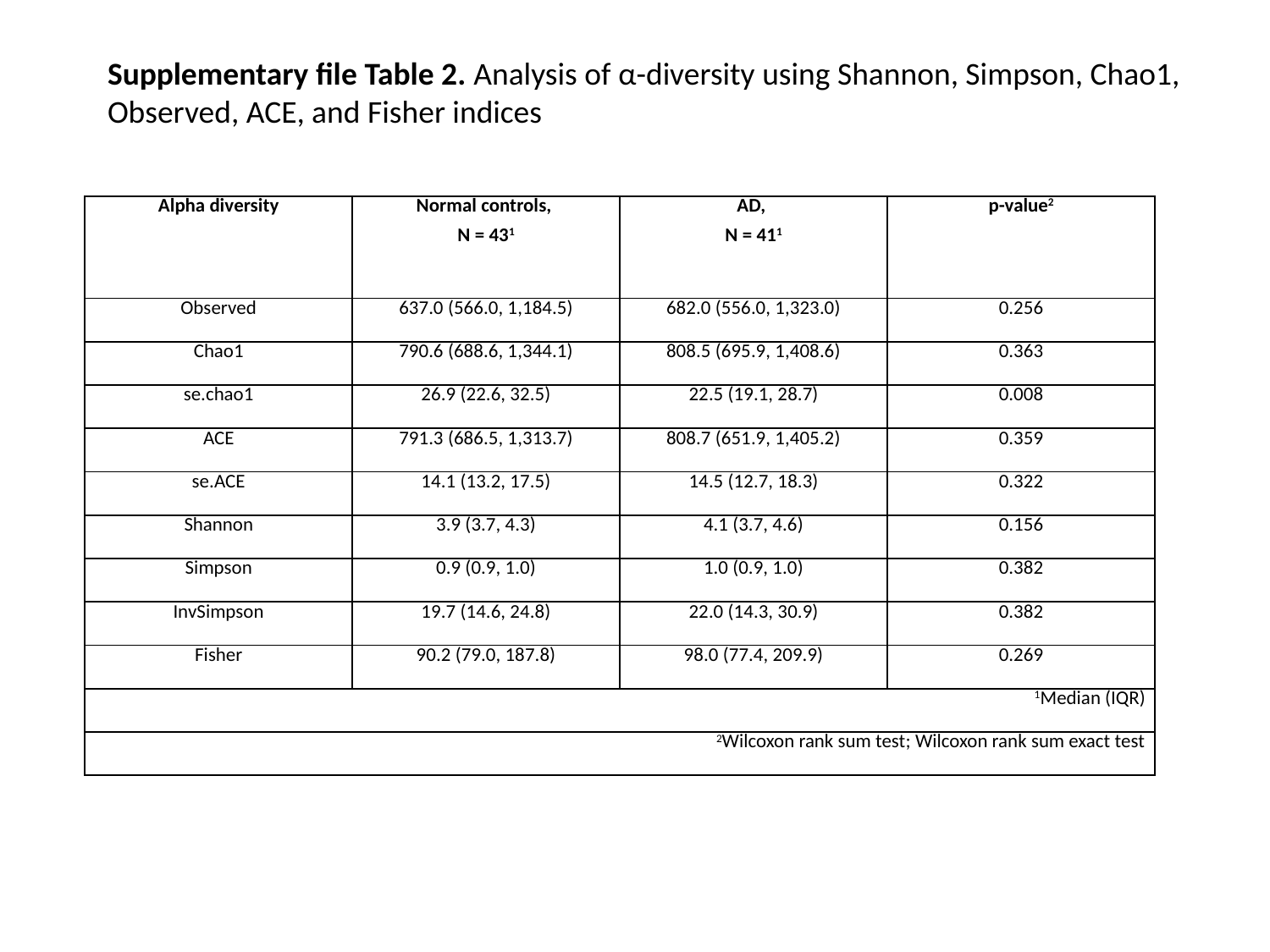

# Supplementary file Table 2. Analysis of α-diversity using Shannon, Simpson, Chao1, Observed, ACE, and Fisher indices
| Alpha diversity | Normal controls, N = 431 | AD, N = 411 | p-value2 |
| --- | --- | --- | --- |
| Observed | 637.0 (566.0, 1,184.5) | 682.0 (556.0, 1,323.0) | 0.256 |
| Chao1 | 790.6 (688.6, 1,344.1) | 808.5 (695.9, 1,408.6) | 0.363 |
| se.chao1 | 26.9 (22.6, 32.5) | 22.5 (19.1, 28.7) | 0.008 |
| ACE | 791.3 (686.5, 1,313.7) | 808.7 (651.9, 1,405.2) | 0.359 |
| se.ACE | 14.1 (13.2, 17.5) | 14.5 (12.7, 18.3) | 0.322 |
| Shannon | 3.9 (3.7, 4.3) | 4.1 (3.7, 4.6) | 0.156 |
| Simpson | 0.9 (0.9, 1.0) | 1.0 (0.9, 1.0) | 0.382 |
| InvSimpson | 19.7 (14.6, 24.8) | 22.0 (14.3, 30.9) | 0.382 |
| Fisher | 90.2 (79.0, 187.8) | 98.0 (77.4, 209.9) | 0.269 |
| 1Median (IQR) | | | |
| 2Wilcoxon rank sum test; Wilcoxon rank sum exact test | | | |

## Slide 6
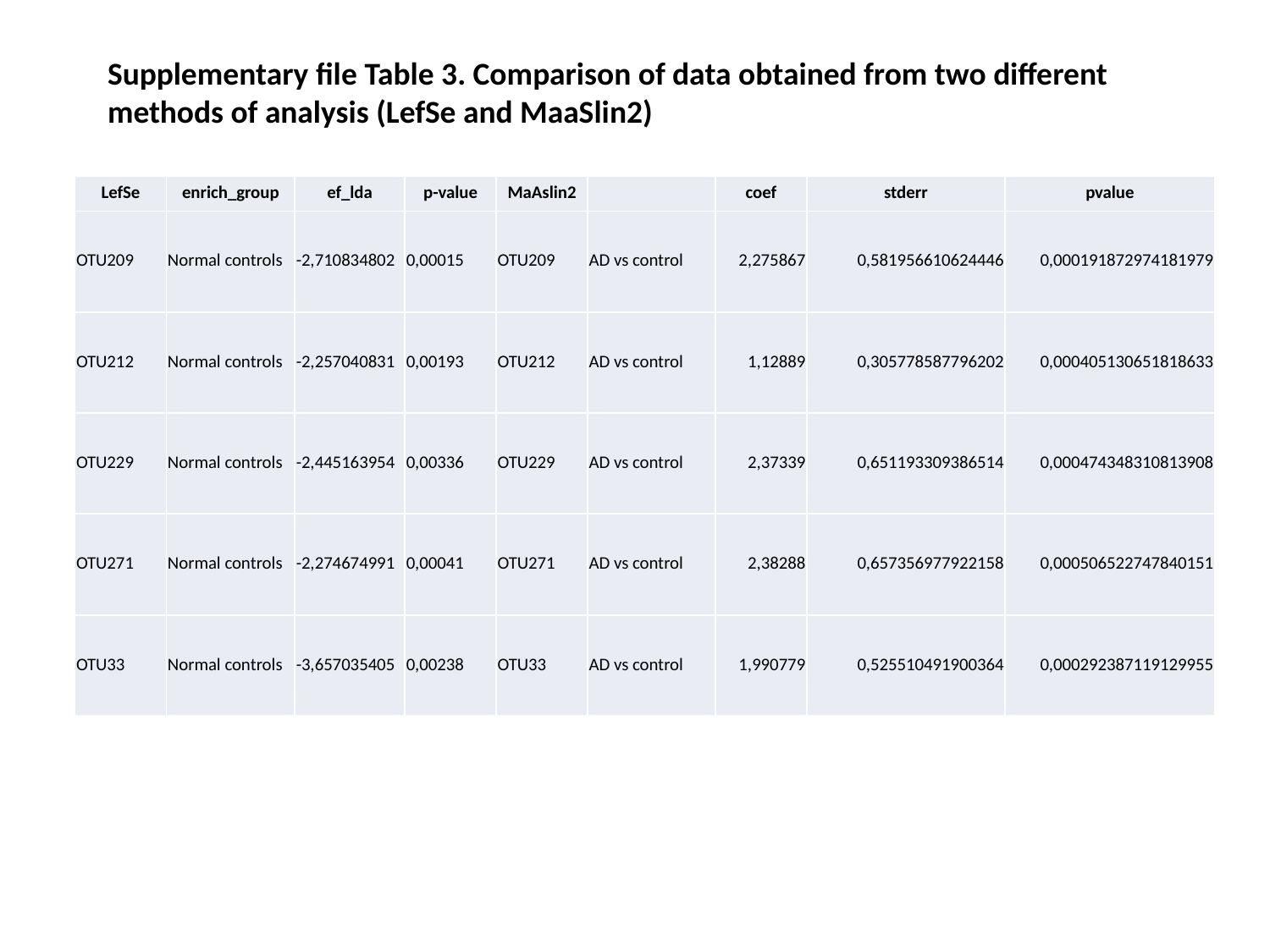

# Supplementary file Table 3. Comparison of data obtained from two different methods of analysis (LefSe and MaaSlin2)
| LefSe | enrich\_group | ef\_lda | p-value | MaAslin2 | | coef | stderr | pvalue |
| --- | --- | --- | --- | --- | --- | --- | --- | --- |
| OTU209 | Normal controls | -2,710834802 | 0,00015 | OTU209 | AD vs control | 2,275867 | 0,581956610624446 | 0,000191872974181979 |
| OTU212 | Normal controls | -2,257040831 | 0,00193 | OTU212 | AD vs control | 1,12889 | 0,305778587796202 | 0,000405130651818633 |
| OTU229 | Normal controls | -2,445163954 | 0,00336 | OTU229 | AD vs control | 2,37339 | 0,651193309386514 | 0,000474348310813908 |
| OTU271 | Normal controls | -2,274674991 | 0,00041 | OTU271 | AD vs control | 2,38288 | 0,657356977922158 | 0,000506522747840151 |
| OTU33 | Normal controls | -3,657035405 | 0,00238 | OTU33 | AD vs control | 1,990779 | 0,525510491900364 | 0,000292387119129955 |

## Slide 7
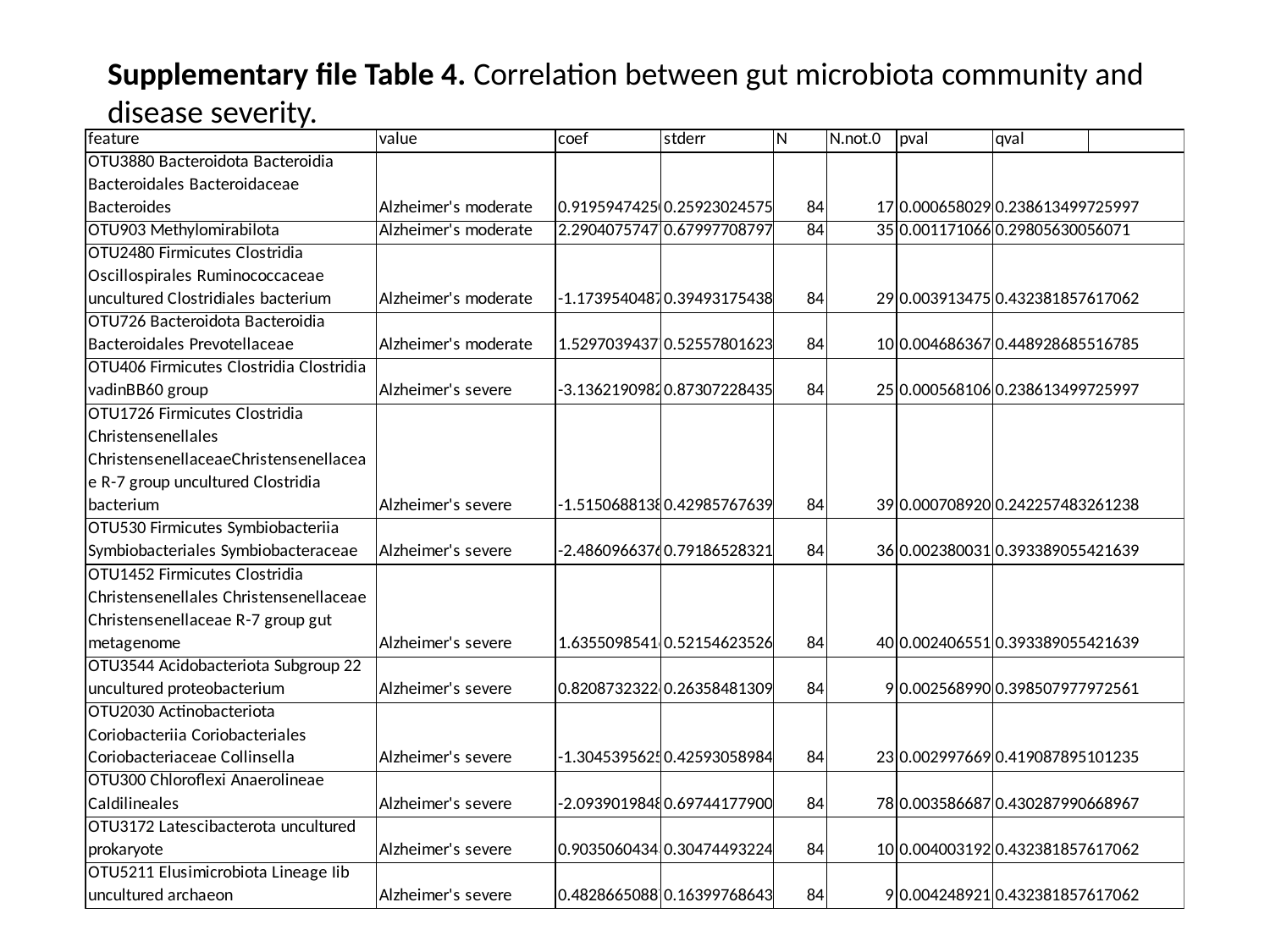

# Supplementary file Table 4. Correlation between gut microbiota community and disease severity.
